# Binge Drinking Among Adults, by Select Characteristics and State — United States, 2018

**DOI:** 10.15585/mmwr.mm7041a2

**Published:** 2021-10-15

**Authors:** Michele K. Bohm, Yong Liu, Marissa B. Esser, Jessica B. Mesnick, Hua Lu, Yi Pan, Kurt J. Greenlund

**Affiliations:** ^1^Division of Population Health, National Center for Chronic Disease Prevention and Health Promotion, CDC; ^2^Division of HIV Prevention, National Center for HIV, Viral Hepatitis, STD, and TB Prevention, CDC.

Excessive alcohol use* is associated with disease, injury, and poor pregnancy outcomes and is responsible for approximately 95,000 deaths in the United States each year (*1*). Binge drinking (five or more drinks on at least one occasion for men or four or more drinks for women) is the most common and costly pattern of excessive alcohol use (*2*). CDC analyzed data from the 2018 Behavioral Risk Factor Surveillance System (BRFSS) to estimate past 30-day binge drinking prevalence, frequency, and intensity (number of drinks per occasion), overall and by select characteristics and state. The overall unadjusted prevalence of binge drinking during the past 30 days was 16.6%, representing an estimated 38.5 million U.S. adults aged ≥18 years; prevalence was highest (26.0%) among those aged 25–34 years. The age-standardized binge drinking prevalence was higher among men (22.5%) than among women (12.6%), increased with income, and was highest among non-Hispanic White adults and adults in the Midwest Census region. State-level age-standardized binge drinking prevalence ranged from 10.5% (Utah) to 25.8% (Wisconsin). Among adults who reported binge drinking, 25.0% did so at least weekly, on average, and 25.0% consumed at least eight drinks on an occasion. To reduce binge drinking, the Community Preventive Services Task Force recommends increasing alcohol taxes and implementing strategies that strengthen regulations to reduce alcohol availability.^†^ The U.S. Preventive Services Task Force recommends clinicians screen adults for alcohol misuse in primary care settings and provide counseling as needed.^§^

BRFSS is an ongoing, state-based, random-digit–dialed, landline and cellular telephone survey of the U.S. noninstitutionalized adult population that collects health-related data nationwide.^¶^ In 2018, the median survey response rate** for all states and the District of Columbia was 49.9% (range = 38.8%–67.2%).^††^ CDC analyzed data from 398,485 respondents aged ≥18 years in the 2018 BRFSS to estimate past 30-day binge drinking prevalence, frequency, and intensity. Binge drinking prevalence and frequency were assessed with the question, “Considering all types of alcoholic beverages, how many times during the past 30 days did you have 5 (4 for women) or more drinks on an occasion?”^§§^ Intensity was assessed with the question, “During the past 30 days, what is the largest number of drinks you had on any occasion?” (*3*). Unadjusted and age-standardized (to the 2000 U.S. standard population) binge drinking prevalence and 95% confidence intervals (CIs) were estimated overall. Age-standardized prevalence was also estimated by respondents’ sociodemographic characteristics (except prevalence by age group), including sex, race/ethnicity, income, marital status, veteran status, education, region, county urbanization level,^¶¶^ and state. State-level prevalence estimates and 95% CIs were grouped into tertiles to identify geographic patterns. Because of the highly right-skewed distribution of the data, similar measures of binge drinking frequency and intensity among adults reporting binge drinking were estimated with medians and variances derived using Taylor series linearization. The means and 75th and 90th percentiles for frequency and intensity were also calculated to further characterize the distributions of these measures. Statistically significant differences between medians were defined as p<0.05 using pairwise tests and nonoverlapping CIs. All analyses were performed using SAS-callable SUDAAN (version 11.0.3; RTI International), and sampling weights were applied to account for complex sampling design, including nonresponse bias and noncoverage errors, and to improve representation of the adult U.S. population in different states.

In 2018, the overall nationwide unadjusted binge drinking prevalence among U.S. adults was 16.6% (95% CI = 16.3%–16.8%), representing an estimated 38.5 million adults (Table 1); prevalence was highest among adults aged 25–34 years (26.0%). Age-standardized binge drinking prevalence was 17.4% (95% CI = 17.2%–17.7%) and varied by sociodemographic group and by state (range = 10.5% [Utah] to 25.8% [Wisconsin]) (Figure) (Supplementary Table, https://stacks.cdc.gov/view/cdc/110373). Binge drinking prevalence was significantly higher among men (22.5%) than among women (12.6%) and was highest among non-Hispanic White adults (19.7%), those with annual household incomes ≥$75,000 (21.4%), those who were never married (18.5%) or were divorced/separated/widowed (19.4%), and veterans (20.9%). Binge drinking prevalence was significantly higher among adults with a college degree (18.9%) than among adults with less than a high school diploma (14.9%). States with higher binge drinking prevalences clustered in the Midwest and Northeast.

**TABLE 1:** Prevalence of binge drinking among adults aged ≥18 years, by selected characteristics — Behavioral Risk Factor Surveillance System, United States,* 2018

Characteristic	Weighted no.^†^ of adults reporting binge drinking,^§^ x 1,000	Binge drinking^§^ prevalence,^¶^ % (95% CI)
**Overall, unadjusted**	**38,544**	**16.6 (16.3–16.8)**
**Overall, age–adjusted**	**NA**	**17.4 (17.2–17.7)**
**Age group, yrs**		
18–24	7,010	24.0 (23.1–24.9)
25–34	10,595	26.0 (25.3–26.7)
35–44	7,579	20.4 (19.7–21.0)
45–64	10,871	14.3 (13.9–14.7)
≥65	2,489	5.0 (4.8–5.3)
**Sex**		
Men	24,603	22.5 (22.0–22.9)
Women	13,941	12.6 (12.2–12.9)
**Race/Ethnicity**		
White, non-Hispanic	25,654	19.7 (19.4–20.0)
Black, non-Hispanic	3,570	13.4 (12.7–14.2)
Hispanic	6,483	16.3 (15.5–17.2)
AI/AN, non-Hispanic	336	16.1 (14.0–18.6)
A/PI, non-Hispanic	1,281	9.7 (8.7–10.8)
Other, non-Hispanic	738	16.9 (15.6–18.2)
**Annual household income**		
<$25,000	7,072	14.6 (14.1–15.2)
$25,000–$49,999	7,545	17.8 (17.1–18.4)
$50,000–$74,999	5,519	19.0 (18.3–19.8)
≥$75,000	14,761	21.4 (20.9–21.9)
**Marital status**		
Married**	19,318	16.6 (16.2–17.0)
Divorced/Separated/Widowed	5,820	19.4 (18.2–20.7)
Never married	13,243	18.5 (17.9–19.1)
**Veteran status**		
Veteran	4,015	20.9 (19.8–21.9)
Nonveteran	34,494	17.1 (16.8–17.4)
**Education level**		
Less than high school graduate	4,116	14.9 (14.0–15.8)
High school graduate or equivalent	10,363	17.2 (16.7–17.8)
Some college	12,862	18.4 (17.9–18.9)
College graduate	11,154	18.9 (18.4–19.3)
**U.S. Census region^††^**		
Northeast	6,742	17.9 (17.3–18.5)
Midwest	9,340	20.0 (19.6–20.5)
South	13,688	16.3 (15.8–16.8)
West	8,775	16.6 (16.1–17.1)
**County urban-rural status^§§^**		
Large central metro	12,298	17.7 (17.1–18.2)
Large fringe metro	9,457	17.3 (16.8–17.9)
Medium metro	7,979	17.5 (17.0–18.0)
Small metro	3,519	17.7 (17.1–18.4)
Micropolitan	3,083	16.7 (16.1–17.4)
Noncore	2,210	16.5 (15.7–17.3)

**Abbreviations:** A/PI = Asian/Pacific Islander; AI/AN = American Indian/Alaska Native; CI = confidence interval; NA = not applicable.

* The study sample included 398,485 adult respondents aged ≥18 years from all 50 states and the District of Columbia with complete information on age, sex, and binge drinking. Weighted numbers were derived through application of survey weights.

^†^ Categories in subgroups might not sum to total because of missing responses for some variables.

^§^ Binge drinking was defined as consuming five or more drinks (men) or four or more drinks (women) on at least one occasion during the past 30 days.

^¶^ Prevalence estimates for all characteristics except age group were age-standardized to the 2000 U.S. standard population.

** Married includes unmarried cohabitating couples.

^††^ Regions were based on U.S. Census Bureau definitions. https://www2.census.gov/geo/pdfs/maps-data/maps/reference/us_regdiv.pdf

^§§^ Counties were classified using the 2013 National Center for Health Statistics Urban-Rural Classification Scheme for Counties. https://www.cdc.gov/nchs/data/series/sr_02/sr02_166.pdf

**FIGURE:**
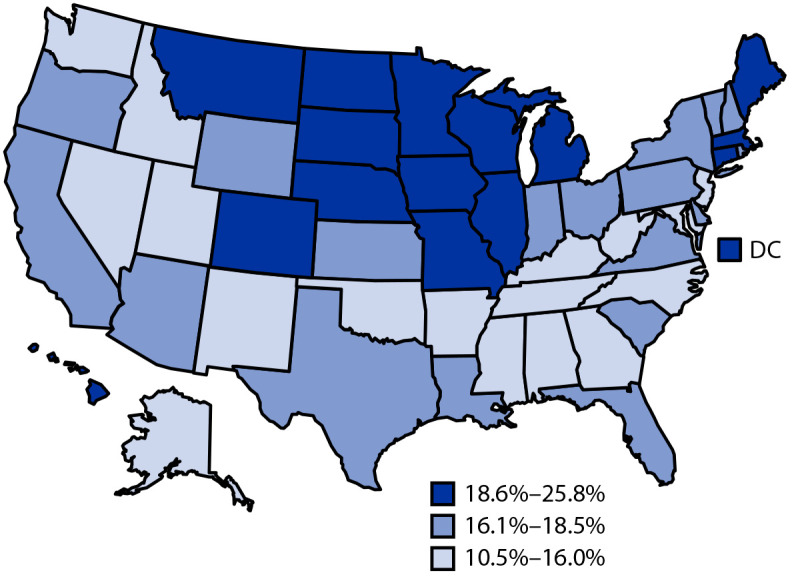
Prevalence of binge drinking* among adults aged ≥18 years — Behavioral Risk Factor Surveillance System, United States,^†^ 2018 **Abbreviation:** DC = District of Columbia. * Respondents who reported consuming five or more alcoholic drinks (men) or four or more alcoholic drinks (women) on at least one occasion in the past 30 days. ^†^ State prevalence estimates are divided into tertiles.

Among adults who reported binge drinking, the median frequency was 1.7 (mean = 4.6) binge drinking occasions during the past 30 days, and the median intensity was 5.5 (mean = 7.2) drinks on an occasion. (Table 2). The upper frequency quartile was >4.0 (95% CI = 3.9–4.1) binge drinking occasions in the past 30 days and the upper intensity quartile was >7.7 (95% CI = 7.6–7.8) drinks on an occasion. Median binge drinking frequency and intensity were significantly higher among men (1.9 occasions and 5.9 drinks, respectively) than among women (1.4 occasions and 4.5 drinks, respectively), and decreased with education level. Median binge drinking intensity was highest among adults aged 18–24 years and decreased with age. Median binge drinking frequency among states ranged from 1.5 occasions (eight states) to 2.1 occasions (Mississippi) in the past 30 days; median binge-drinking intensity on an occasion ranged from 5.2 drinks (New Jersey, District of Columbia, and Connecticut) to 6.4 drinks (West Virginia).

**TABLE 2:** Binge drinking frequency and intensity among adults aged ≥18 years who binge drank in the past 30 days, by selected characteristics and state — Behavioral Risk Factor Surveillance System, United States,* 2018

Characteristic	Adults who binge drank in the past 30 days					
	Binge drinking^†^ frequency^§^ (n = 54,045)			Binge drinking^†^ intensity^¶^ (n = 50,527)		
	No. of occasions** (95% CI)			No. of drinks** (95% CI)		
	Median	75th percentile	90th percentile	Median	75th percentile	90th percentile
**Overall, unadjusted**	**1.7 (1.7–1.7)**	**4.0 (3.9–4.1)**	**9.5 (9.4–9.7)**	**5.5 (5.5–5.5)**	**7.7 (7.6–7.8)**	**11.5 (11.4–11.6)**
**Age group, yrs**						
18–24	1.7 (1.6–1.8)	4.1 (3.9–4.4)	9.1 (7.7–9.4)	5.9 (5.8–6.0)	9.2 (8.9–9.3)	13.6 (12.0–14.3)
25–34	1.6 (1.5–1.7)	3.8 (3.6–3.9)	9.1 (7.8–9.4)	5.7 (5.7–5.8)	8.9 (8.2–9.1)	11.9 (11.7–12.3)
35–44	1.7 (1.6–1.8)	3.9 (3.8–4.1)	9.7 (9.4–11.1)	5.5 (5.4–5.6)	7.6 (7.4–7.8)	11.4 (11.2–11.6)
45–64	1.8 (1.7–1.9)	4.3 (4.0–4.4)	10.0 (9.7–11.9)	5.3 (5.2–5.3)	7.0 (6.7–7.2)	11.1 (9.9–11.3)
≥65	1.8 (1.6–1.9)	4.4 (4.1–4.7)	14.2 (9.9–18.5)	5.0 (4.9–5.0)	5.8 (5.7–5.8)	7.9 (7.4– 9.1)
**Sex**						
Men	1.9 (1.8–1.9)	4.6 (4.4–4.7)	11.4 (10.0–12.0)	5.9 (5.9–6.0)	9.3 (9.2–9.4)	12.7 (12.0–13.8)
Women	1.4 (1.3–1.5)	3.3 (3.2–3.4)	6.8 (6.2–7.3)	4.5 (4.5–4.5)	5.6 (5.5–5.7)	7.9 (7.7– 8.6)
**Education level**						
Less than high school graduate	2.3 (2.0–2.6)	4.9 (4.5–5.8)	19.1 (13.6–24.9)	5.8 (5.7–5.9)	9.7 (9.3–10.0)	14.8 (14.1–17.2)
High school graduate or equivalent	1.9 (1.8–2.0)	4.6 (4.4–4.8)	11.6 (10.0–13.9)	5.7 (5.6–5.7)	9.0 (8.4–9.2)	11.9 (11.8–12.9)
Some college	1.7 (1.6–1.8)	4.0 (3.9–4.2)	9.5 (9.2–9.7)	5.5 (5.5–5.6)	7.7 (7.5–7.8)	11.4 (11.2–11.5)
College graduate	1.4 (1.3–1.4)	3.3 (3.1–3.4)	6.9 (6.2–7.3)	5.2 (5.2–5.3)	6.8 (6.6–6.9)	9.6 (9.5– 9.7)
**Race/Ethnicity**						
White, non-Hispanic	1.7 (1.7–1.8)	4.1 (4.0–4.2)	9.7 (9.5–9.8)	5.5 (5.5–5.6)	7.7 (7.6–7.8)	11.5 (11.4–11.6)
Black, non-Hispanic	1.8 (1.7–2.0)	4.2 (3.8–4.6)	9.7 (8.4–12.3)	5.1 (5.0–5.3)	6.3 (6.0–6.7)	9.7 (9.3–11.1)
Hispanic	1.6 (1.5–1.7)	3.7 (3.5–3.9)	7.7 (7.1–9.3)	5.7 (5.6–5.8)	8.9 (7.8–9.2)	11.8 (11.5–12.0)
AI/AN, non-Hispanic	2.2 (1.8–2.9)	5.5 (4.4–7.2)	18.8 (14.1–25.0)	5.7 (5.4–6.0)	9.1 (7.4–9.7)	13.0 (11.5–14.7)
A/PI, non-Hispanic	1.4 (1.2–1.6)	3.3 (2.7–3.9)	7.2 (5.1–9.4)	5.3 (5.1–5.5)	6.8 (6.2–7.4)	9.9 (9.4–11.3)
Other, non-Hispanic	1.9 (1.7–2.2)	4.7 (3.9–5.7)	11.4 (9.5–14.4)	5.7 (5.5–5.8)	8.3 (7.7–9.3)	12.7 (11.5–14.7)
**Annual household income**						
<$25,000	1.9 (1.8–2.0)	4.5 (4.2–4.7)	14.2 (11.4–14.8)	5.5 (5.5–5.6)	8.0 (7.7–9.0)	11.8 (11.6–12.0)
$25,000–$49,999	1.7 (1.6–1.8)	4.0 (3.8–4.3)	9.7 (9.3–10.0)	5.5 (5.5–5.6)	7.8 (7.5–8.1)	11.6 (11.4–11.9)
$50,000–$74,999	1.8 (1.7–1.9)	4.2 (3.9–4.5)	9.5 (9.1–9.9)	5.6 (5.6–5.7)	8.0 (7.8–9.0)	11.7 (11.5–11.9)
≥$75,000	1.6 (1.5–1.7)	3.8 (3.7–3.9)	9.1 (7.8–9.3)	5.5 (5.4–5.5)	7.5 (7.3–7.6)	11.2 (11.1–11.4)
**Marital status**						
Married^††^	1.6 (1.5–1.6)	3.7 (3.6–3.8)	9.2 (8.0–9.4)	5.4 (5.3–5.4)	7.2 (7.1–7.3)	11.0 (10.0–11.2)
Divorced/Separated/ Widowed	1.9 (1.8–2.0)	4.8 (4.5–5.2)	18.2 (14.5–19.9)	5.4 (5.3–5.5)	7.6 (7.3–7.8)	11.6 (11.4–11.9)
Never married	1.8 (1.7–1.9)	4.2 (4.0–4.4)	9.3 (9.0–9.5)	5.8 (5.7–5.9)	9.1 (8.8–9.2)	12.0 (11.8–12.7)
**Veteran status**						
Veteran	1.9 (1.7–2.0)	4.6 (4.2–4.9)	14.3 (10.9–15.4)	5.7 (5.6–5.8)	7.9 (7.6–9.0)	11.7 (11.4–11.9)
Nonveteran	1.7 (1.6–1.7)	4.0 (3.9–4.1)	9.4 (9.2–9.6)	5.5 (5.4–5.5)	7.7 (7.6–7.8)	11.5 (11.4–11.6)
**County urban-rural status^§§^**						
Large central metro	1.6 (1.6–1.7)	3.9 (3.7–4.0)	9.2 (8.0–9.6)	5.5 (5.4–5.5)	7.5 (7.3–7.7)	11.3 (11.0–11.5)
Large fringe metro	1.6 (1.5–1.7)	3.8 (3.6–4.0)	9.0 (7.7–9.4)	5.4 (5.4–5.5)	7.5 (7.3–7.7)	11.3 (11.1–11.5)
Medium metro	1.7 (1.7–1.8)	4.2 (4.0–4.4)	9.7 (9.4–10.0)	5.5 (5.5–5.6)	7.7 (7.5–7.9)	11.5 (11.3–11.7)
Small metro	1.9 (1.8–2.0)	4.4 (4.1–4.6)	9.9 (9.4–11.9)	5.6 (5.6–5.7)	9.0 (7.9–9.2)	11.8 (11.5–12.0)
Micropolitan	1.8 (1.7–1.9)	4.5 (4.2–4.8)	11.5 (9.8–14.1)	5.6 (5.5–5.7)	8.3 (7.8–9.1)	11.9 (11.7–14.1)
Noncore	1.9 (1.8–2.1)	4.5 (4.1–4.8)	12.0 (10.0–14.3)	5.7 (5.6–5.8)	9.1 (8.3–9.3)	12.1 (11.8–14.3)
**U.S. Census region** ^¶¶^						
Northeast	1.6 (1.5–1.7)	3.7 (3.6–3.9)	7.7 (7.3–9.1)	5.4 (5.3–5.4)	7.3 (7.1–7.5)	11.0 (9.9–11.3)
Midwest	1.8 (1.7–1.8)	4.2 (4.0–4.4)	9.7 (9.5–10.0)	5.6 (5.5–5.7)	8.0 (7.9–8.7)	11.8 (11.6–11.9)
South	1.8 (1.7–1.8)	4.3 (4.1–4.6)	10.1 (9.7–11.8)	5.5 (5.5–5.6)	7.7 (7.5–7.9)	11.5 (11.4–11.7)
West	1.6 (1.5–1.7)	3.8 (3.6–3.9)	9.1 (7.8–9.4)	5.5 (5.5–5.6)	7.7 (7.5–7.8)	11.4 (11.2–11.6)
**State**						
Alabama	1.7 (1.5–1.9)	4.0 (3.4–4.6)	8.4 (6.7–9.7)	5.5 (5.3–5.6)	7.2 (6.5–8.0)	10.8 (9.8–11.9)
Alaska	1.9 (1.4–2.7)	4.4 (3.7–5.1)	9.6 (5.8–14.1)	5.4 (5.2–5.6)	7.1 (6.0–7.6)	9.6 (7.9–11.4)
Arizona	1.5 (1.3–1.7)	3.8 (3.2–4.3)	9.2 (7.2–9.8)	5.5 (5.4–5.7)	7.9 (7.3–9.1)	11.3 (9.9–11.8)
Arkansas	1.9 (1.6–2.3)	4.5 (3.8–5.5)	11.2 (9.3–14.6)	5.6 (5.4–5.8)	9.2 (7.4–9.7)	11.8 (11.3–14.0)
California	1.6 (1.4–1.7)	3.7 (3.4–3.9)	7.9 (7.2–9.4)	5.5 (5.4–5.6)	7.7 (7.4–8.2)	11.5 (11.2–11.8)
Colorado	1.6 (1.4–1.7)	3.7 (3.4–4.0)	9.1 (7.3–9.6)	5.6 (5.5–5.7)	7.6 (7.3–7.8)	11.4 (9.9–11.8)
Connecticut	1.6 (1.4–1.7)	3.5 (3.1–3.8)	7.8 (5.9–9.5)	5.2 (5.1–5.3)	6.5 (6.0–6.9)	9.3 (8.0– 9.8)
Delaware	1.7 (1.5–2.0)	4.4 (3.8–5.2)	11.3 (8.4–14.6)	5.3 (5.0–5.5)	7.3 (6.6–7.8)	11.3 (9.6–11.9)
District of Columbia	1.5 (1.3–1.7)	3.4 (2.9–4.0)	6.9 (5.5–7.9)	5.2 (5.0–5.4)	6.7 (6.0–7.2)	9.4 (8.1– 9.8)
Florida	1.8 (1.6–2.0)	4.9 (4.1–5.8)	14.2 (9.7–19.7)	5.5 (5.3–5.6)	7.3 (6.9–7.7)	11.1 (9.7–11.7)
Georgia	1.6 (1.5–1.8)	4.1 (3.7–4.5)	9.7 (8.3–13.9)	5.3 (5.1–5.5)	7.5 (7.1–7.9)	11.5 (11.0–11.8)
Hawaii	1.9 (1.7–2.2)	4.3 (3.9–4.7)	11.1 (8.8–14.3)	5.7 (5.5–5.8)	9.2 (7.9–9.6)	13.3 (11.5–14.8)
Idaho	2.0 (1.7–2.5)	4.5 (3.9–5.3)	10.5 (8.0–17.9)	5.8 (5.6–6.1)	9.1 (7.7–9.7)	13.1 (11.4–14.4)
Illinois	1.9 (1.7–2.1)	4.4 (3.9–4.8)	9.7 (7.9–11.9)	5.7 (5.5–5.8)	9.1 (7.8–9.5)	14.0 (11.7–14.6)
Indiana	1.7 (1.5–1.9)	4.2 (3.7–4.8)	9.9 (9.2–14.5)	5.6 (5.4–5.8)	9.1 (7.8–9.6)	14.1 (11.7–15.5)
Iowa	1.9 (1.8–2.0)	4.3 (4.0–4.5)	9.5 (9.1–9.9)	5.8 (5.7–5.9)	9.2 (8.8–9.4)	11.9 (11.7–12.7)
Kansas	1.6 (1.4–1.7)	3.8 (3.5–4.1)	8.6 (7.1–9.8)	5.6 (5.5–5.7)	7.8 (7.5–9.0)	11.5 (11.1–11.9)
Kentucky	1.9 (1.7–2.4)	5.0 (4.2–6.9)	10.2 (9.6–14.4)	5.7 (5.5–5.9)	8.8 (7.5–9.4)	13.7 (11.4–14.8)
Louisiana	2.0 (1.7–2.4)	4.7 (4.2–5.5)	9.9 (9.1–14.1)	5.5 (5.3–5.6)	7.4 (6.7–8.0)	11.3 (9.8–11.9)
Maine	1.8 (1.6–2.0)	4.1 (3.6–4.7)	9.3 (7.3–11.4)	5.4 (5.2–5.5)	7.1 (6.5–7.8)	11.2 (9.6–11.6)
Maryland	1.6 (1.4–1.8)	3.7 (3.4–4.0)	7.9 (6.7–9.7)	5.3 (5.2–5.5)	7.0 (6.6–7.3)	10.0 (9.6–11.5)
Massachusetts	1.5 (1.4–1.7)	3.6 (3.2–3.9)	7.0 (5.4–7.9)	5.4 (5.2–5.5)	7.0 (6.5–7.6)	11.2 (9.8–11.6)
Michigan	1.8 (1.6–1.9)	4.3 (3.9–4.7)	10.0 (9.4–14.0)	5.6 (5.4–5.7)	7.9 (7.6–9.0)	11.7 (11.2–14.0)
Minnesota	1.5 (1.4–1.6)	3.7 (3.5–3.9)	9.1 (7.4–9.4)	5.5 (5.4–5.6)	7.7 (7.5–7.9)	11.3 (11.0–11.6)
Mississippi	2.1 (1.8–2.7)	5.4 (4.1–8.8)	14.8 (10.0–22.1)	5.7 (5.5–5.9)	9.4 (7.8–10.3)	14.6 (11.3–17.4)
Missouri	1.8 (1.6–2.2)	4.8 (4.1–6.1)	14.0 (9.5–14.9)	5.5 (5.3–5.7)	7.7 (7.1–9.0)	11.7 (11.2–12.5)
Montana	1.6 (1.4–1.8)	4.0 (3.5–4.5)	9.1 (6.9–14.3)	5.4 (5.3–5.6)	7.3 (6.8–7.8)	10.4 (9.6–11.5)
Nebraska	1.7 (1.6–1.8)	3.9 (3.6–4.3)	9.1 (7.5–9.7)	5.6 (5.5–5.7)	8.0 (7.6–9.1)	11.4 (11.1–11.7)
Nevada	1.6 (1.3–1.9)	3.7 (2.9–4.5)	7.4 (5.6–9.3)	5.5 (5.2–5.7)	7.8 (6.7–9.3)	11.2 (9.8–14.1)
New Hampshire	1.5 (1.3–1.8)	4.0 (3.4–4.7)	9.9 (7.7–13.2)	5.5 (5.3–5.7)	7.6 (6.9–8.3)	11.2 (9.5–11.7)
New Jersey	—***	3.5 (2.9–3.9)	7.2 (4.7–9.6)	5.2 (4.9–5.6)	7.3 (6.3–9.1)	9.7 (9.4– 9.9)
New Mexico	1.8 (1.5–2.1)	4.5 (4.0–5.2)	11.4 (9.5–17.8)	5.5 (5.3–5.7)	7.9 (7.2–9.4)	13.1 (11.5–18.3)
New York	1.6 (1.5–1.7)	3.7 (3.4–4.0)	7.7 (6.9–9.2)	5.4 (5.3–5.5)	7.1 (6.8–7.4)	11.0 (9.8–11.4)
North Carolina	1.8 (1.5–2.2)	4.6 (3.9–5.5)	10.7 (7.7–14.6)	5.4 (5.3–5.6)	7.0 (6.4–7.7)	10.5 (9.7–11.3)
North Dakota	1.8 (1.5–2.0)	4.2 (3.7–4.7)	8.2 (6.9–9.9)	5.9 (5.7–6.3)	9.4 (9.1–9.7)	14.1 (11.7–14.6)
Ohio	1.9 (1.7–2.2)	4.4 (3.9–4.9)	12.0 (9.5–18.6)	5.7 (5.5–5.8)	8.2 (7.7–9.2)	11.8 (11.3–13.6)
Oklahoma	1.5 (1.2–1.7)	3.8 (3.0–4.5)	9.3 (7.3–16.0)	5.6 (5.4–5.8)	7.9 (7.2–9.3)	11.5 (9.9–12.0)
Oregon	1.7 (1.5–1.9)	4.1 (3.6–4.5)	9.6 (8.3–13.2)	5.5 (5.4–5.6)	7.4 (6.9–7.8)	9.9 (9.6–11.4)
Pennsylvania	1.8 (1.5–2.0)	3.9 (3.6–4.4)	7.9 (7.0–10.1)	5.5 (5.3–5.6)	7.9 (7.4–9.1)	11.5 (10.0–12.7)
Rhode Island	1.6 (1.4–1.8)	3.8 (3.1–4.4)	8.5 (6.5–9.9)	5.4 (5.1–5.6)	7.2 (6.7–7.7)	9.7 (9.2–10.5)
South Carolina	1.9 (1.7–2.2)	4.6 (4.1–5.2)	11.4 (9.5–14.9)	5.7 (5.5–5.8)	8.4 (7.7–9.3)	11.7 (11.2–12.0)
South Dakota	1.7 (1.4–2.1)	4.0 (3.5–4.6)	7.8 (5.9–9.3)	5.7 (5.5–5.9)	7.9 (7.4–9.2)	11.6 (9.9–12.8)
Tennessee	1.7 (1.4–2.1)	4.4 (3.6–5.2)	9.2 (6.8–12.2)	5.5 (5.3–5.7)	7.6 (6.9–9.1)	11.8 (11.2–14.4)
Texas	1.8 (1.5–2.0)	4.1 (3.6–4.9)	9.9 (7.6–14.0)	5.6 (5.4–5.8)	9.0 (7.6–9.5)	11.7 (11.2–14.2)
Utah	1.7 (1.6–1.9)	4.4 (4.0–4.8)	10.2 (8.9–14.4)	5.6 (5.4–5.7)	7.8 (7.4–8.7)	11.4 (10.3–14.1)
Vermont	1.9 (1.6–2.3)	4.3 (3.7–5.1)	9.5 (8.1–12.3)	5.5 (5.2–5.7)	7.5 (7.0–8.0)	10.6 (9.8–11.6)
Virginia	1.6 (1.5–1.8)	3.7 (3.4–4.0)	9.8 (9.1–14.0)	5.5 (5.3–5.6)	7.2 (6.8–7.7)	11.3 (10.0–11.6)
Washington	1.5 (1.4–1.7)	3.6 (3.3–3.9)	9.2 (7.0–9.8)	5.3 (5.1–5.4)	6.8 (6.3–7.2)	9.9 (9.5–11.3)
West Virginia	1.8 (1.5–2.4)	5.1 (4.3–7.1)	15.2 (11.5–20.8)	6.4 (5.9–7.2)	9.8 (9.2–11.3)	14.6 (12.0–17.5)
Wisconsin	1.7 (1.5–1.9)	3.8 (3.4–4.3)	9.1 (6.6–9.7)	5.5 (5.4–5.7)	7.5 (7.0–7.9)	10.8 (9.8–11.4)
Wyoming	1.5 (1.3–1.7)	4.1 (3.5–4.6)	10.4 (8.3–14.3)	5.5 (5.4–5.7)	7.5 (6.8–8.8)	11.6 (11.0–12.5)

**Abbreviations:** A/PI = Asian/Pacific Islander; AI/AN = American Indian/Alaska Native; CI = confidence interval.

* The study sample included adult respondents aged ≥18 years from all 50 states and the District of Columbia with complete information on age, sex, and binge drinking and who reported binge drinking.

^†^ Binge drinking was defined as consuming five or more drinks (men) or four or more drinks (women) on at least one occasion during the past 30 days.

^§^ Number of binge drinking occasions in the past 30 days among adults who reported binge drinking.

^¶^ Largest number of drinks consumed on any occasion in the past 30 days among adults who reported binge drinking.

** Unadjusted weighted medians and percentiles were derived from SAS-callable SUDAAN “proc descript”; variance estimates were derived using the Taylor series linearization method.

^††^ Married includes unmarried cohabitating couples.

^§§^ Counties were classified using the 2013 National Center for Health Statistics Urban–Rural Classification Scheme for Counties. https://www.cdc.gov/nchs/data/series/sr_02/sr02_166.pdf

^¶¶^ Regions were based on U.S. Census Bureau definitions. https://www2.census.gov/geo/pdfs/maps-data/maps/reference/us_regdiv.pdf

*** Estimates are unreliable if the relative standard error is >0.3.

## Discussion

During 2018, one in six U.S. adults reported binge drinking during the past 30 days, increasing their risk for many preventable adverse health outcomes. Among those who binge drank, one half did so at least twice per month; one half of men consumed at least six drinks and one half of women consumed at least five drinks on a binge occasion. These median values are lower than the mean values for binge drinking frequency and intensity, but better represent how often adults who binge drink typically do so and how many drinks they usually consume. The higher values for the 90th percentiles for frequency (9.5 occasions in the past 30 days) and intensity (11.5 drinks on an occasion) indicate that a small proportion of adults binge drink very frequently, consume large quantities of alcohol, or both, which is consistent with previous findings (*4*).

Binge drinking prevalence decreased from 18.9% in 2011 to 18.0% in 2017 (*5*). This report found binge drinking prevalence was 17.4% in 2018, indicating that binge drinking remained common. Alcohol consumption patterns might have since changed, including during the COVID-19 pandemic. Collectively, all three measures (prevalence, frequency, and intensity) address a complex pattern of binge drinking. For example, lower education and income levels were associated with lower binge drinking prevalence, but among adults who reported binge drinking, those with less than a high school diploma reported higher frequency and intensity than did college graduates. Similarly, adults in the lowest income level binge drank more frequently than did adults in the highest income level. The finding that the prevalence of binge drinking was lower in the most rural counties than in the most urban counties is consistent with earlier reports (*6*). However, adults in the most rural counties who binge drank did so more frequently and at higher intensity than did adults in the most urban counties. The prevalence of binge drinking in Mississippi and in West Virginia was lower than in the United States overall, but Mississippi had the highest median frequency and West Virginia had the highest median intensity of binge drinking among all states.

Excessive alcohol use is associated with increasing mortality from alcoholic liver disease, which has contributed to recently observed declines in U.S. life expectancy, notably among men, young and middle-aged adults, and persons with less than a high school education and limited income living in rural areas (*7*). The results of this study highlight the importance of reducing binge drinking, particularly among groups who are disproportionately affected.

The findings in this report are subject to at least three limitations. First, the BRFSS response rate indicates the potential for selection bias to the extent that survey respondents differ from nonrespondents. Second, responses are self-reported and subject to recall, social desirability, and nonresponse biases, which could vary across states and groups, and lead to underestimates of binge drinking (*8*). A study comparing BRFSS estimates to alcohol sales data found that although they were consistently correlated, survey data substantially underestimated consumption (*9*). Finally, binge drinking intensity based on the largest number of drinks reported on any occasion in the past 30 days might overestimate intensity. A previous analysis found that among demographic groups, this measure was 0.1–1.2 drinks higher than the reported number of drinks consumed during the most recent binge, but the two measures were strongly correlated (*3*). However, they were not correlated among adults without a high school diploma; in 2018, intensity by education level was highest among this group.

A population health approach has been shown to reduce excessive drinking, including binge drinking. The Community Preventive Services Task Force recommends the following strategies to reduce excessive drinking: increasing alcohol taxes, limiting hours and days of alcohol sales, and regulating alcohol outlet density. Fewer than one half of adults who report binge drinking to a health care provider during a medical checkup are advised to reduce their drinking (*10*). Clinicians should follow the U.S. Preventive Services Task Force recommendation to screen all adults for alcohol misuse and provide brief intervention and referral to treatment as needed.

**Summary**

**What is already known about this topic?**

Excessive alcohol use has contributed to declines in life expectancy. Binge drinking is a common and costly pattern of excessive alcohol use.

**What is added by this report?**

During 2018, one in six U.S. adults reported binge drinking during the past 30 days. Among those who binge drank, 25% did so at least weekly, on average, and 25% consumed at least eight drinks during a binge occasion. Some sociodemographic groups and states with low binge drinking prevalence reported large quantities of alcohol consumed during binge occasions.

**What are the implications for public health practice?**

An effective population health approach including regulating alcohol sales, increasing alcohol taxes, and alcohol screening and brief counseling by clinicians can help reduce binge drinking.

## Footnotes

* Excessive alcohol use includes binge drinking, heavy drinking (i.e., 15 or more drinks per week for men; eight or more drinks per week for women), and any drinking by pregnant women or persons aged <21 years. https://www.cdc.gov/alcohol/fact-sheets/alcohol-use.htm

†
https://www.thecommunityguide.org/topic/excessive-alcohol-consumption

§
https://www.uspreventiveservicestaskforce.org/uspstf/recommendation/unhealthy-alcohol-use-in-adolescents-and-adults-screening-and-behavioral-counseling-interventions

¶
https://www.cdc.gov/brfss/annual_data/2018/pdf/overview-2018-508.pdf

** Response rates for BRFSS are calculated using standards set by the American Association for Public Opinion Research. https://www.aapor.org/Standards-Ethics/Standard-Definitions-(1).aspx

††
https://www.cdc.gov/brfss/annual_data/2018/pdf/2018-sdqr-508.pdf

§§
https://www.cdc.gov/brfss/questionnaires/pdf-ques/2018_BRFSS_English_Questionnaire.pdf

¶¶
https://www.cdc.gov/nchs/data_access/urban_rural.htm
